# Fit one model, report one number: how to analyse a training or intervention study

**DOI:** 10.3389/fphys.2026.1949947

**Published:** 2026-09-18

**Authors:** Gudrun Schappacher-Tilp, Markus Tilp

**Affiliations:** 1 Department of Engineering, FH JOANNEUM - University of Applied Sciences, Graz, Austria; 2 Department of Human Movement Science, Sport and Health, University of Graz, Graz, Austria

**Keywords:** analysis of covariance, estimand, generalized linear models, interaction contrasts, repeated measures, replicability, scale dependence

## Abstract

A typical training or intervention study allocates participants to protocols, measures them repeatedly over time, and asks whether one protocol changes the outcome more than another. A typical analysis, such as an analysis of variance or a mixed model, is powerful, but that power is for taking a measurement apart, not for delivering the verdict. Such a model splits each measurement into separate terms: a benchmark, covariate adjustments, a time course, and a term that lets each protocol follow its own time course. The statistical software prints a separate test for every one of these terms, and a common workflow reports those tests as the study’s result. However, the study was built to answer only a single question, usually whether one protocol improves the outcome more than another as training goes on. Our position follows directly: choose that one comparison in advance and test it directly, reading it off the fitted model without first requiring an overall, or omnibus, test to pass. This approach can recover effects the usual workflow can miss, because requiring an overall test to pass first costs a substantial amount of power, as our worked examples show. An overall test remains the right test only when the overall question is the one the study asked. Fit the model as richly as the design demands, then report the one number it was built to produce.

## Introduction

Sport and exercise science has begun to audit itself: replicated effects are far smaller than the published originals, and poor statistical reporting often blocks replication outright (Murphy et al., 2025; Kowalski, 2026). Replication presupposes a single stated quantity the original study claimed to estimate. However, many studies report whichever model term reached significance, and that is not a stated quantity. Design is the larger lever (Mesquida et al., 2025), but a good study can still be wasted at the last step, where the model is fitted and one number should be read off it.

The models are familiar. Sport and exercise science relies heavily on factorial and repeated-measures designs: participants are allocated to training protocols, measured repeatedly over time, and compared on outcomes such as maximal oxygen uptake ( $\dot{V}O_{2}max$), jump height, or a discrete (yes/no) responder status. Analysis of variance (ANOVA), analysis of covariance (ANCOVA), and the wider class of generalised linear and mixed models (Nelder and Wedderburn, 1972; McCullagh and Nelder, 1989) are the standard tools. It is worth recalling what such a model does. It decomposes a measurement, much as a prism splits white light into separate colours. It does not test it. A two-factor repeated-measures ANOVA splits each observation into five pieces: a benchmark that all groups share, how far each protocol sits from it, how far each timepoint sits from it, a term that lets each protocol follow its own time course, and how far each participant sits from the average of that protocol, with whatever is left over as the residual. The algebra is provided in **Supplementary Section S1**.

In a companion paper, we argued that whether to use an omnibus test, that is, a single overall test of a whole model term at once, or to correct for multiplicity should follow the inferential goal, not the number of tests performed (Schappacher-Tilp and Tilp, 2026), echoing a longer argument in biostatistics and epidemiology (Rothman, 1990; Bender and Lange, 2001). There we distinguished three inferential modes, set out in **Supplementary Table S1**: directed testing of a single prespecified effect, localisation across many candidates under false discovery rate control, that is, searching for where an effect is rather than asking one question about it, and the genuinely global question, for which the omnibus test is the right tool.

The present Perspective concerns studies in which the scientific question is fixed before the data are analysed. Exploratory work, where the aim is to find out which effects exist at all, has different goals and lies outside our scope.

The advice to test the comparison the study was designed to make has been in the textbooks for decades (Rosenthal et al., 2000; Wilkinson and Task Force on Statistical Inference, 1999). However, those textbooks describe a simpler study than many of us now run. With one factor and a few groups, the comparison you want is easy to get. Add covariates, several timepoints, and repeated measurements on the same people, and it is nowhere in the output: the software prints one test for each part of the model, none of them is the answer, and you have to ask for it. That gap, between advice written for simple designs and software written for complicated ones, is what we address. We also put a number on what the habit costs. Furthermore, we add one point that earlier treatments did not need to address: for a discrete outcome, an effect can look large or small depending on how it is expressed, so the study has to state the scale on which the effect is being claimed.

## The pipeline in the wild

A common analytical workflow begins with omnibus tests before examining the comparisons that motivated the study. Participants are allocated to either a control condition or training protocols such as moderate-intensity continuous training (MICT) and high-intensity interval training (HIIT), and measured repeatedly over time. The full factorial model is fitted, the terms that reach significance are noted, *post-hoc* pairwise comparisons are run on those terms, and the comparisons that reach significance are reported. This ordering matches the default software output; it carries the reassuring air of a gate that guards against false positives, and it is what the majority of us were taught and have seen in print for decades. Our view is that this habit quietly works against the study’s own goal in three ways. First, the gate is the most diffuse test available, so the loss of power is at its most severe. Second, following up only the terms that were significant is selection on the data: the same measurements choose the hypotheses and then test them, which biases the surviving estimates upward and invalidates their nominal *p*-values (Kriegeskorte et al., 2009). Third, the *post-hoc* comparisons are generic rather than the single targeted comparison the study was built around, so the study pays a multiplicity penalty on comparisons no one cared about, whilst the question it was designed to answer goes untested. Where each of these steps costs more than it appears, and what to do instead, is clearest once we look at what the model is actually estimating.

## What the study is actually asking

Go back to HIIT against control. Suppose the question is whether HIIT increases $\dot{V}O_{2}max$ more than the control condition does from baseline to week 12. Only one combination of the model’s pieces answers it, and that combination is the target comparison: the gain under HIIT minus the gain under control over those 12 weeks, a difference of two differences. This is the quantity a study is designed to estimate, fixed by the scientific question itself and not by whatever model is later chosen to estimate it. Reporting guidelines call it the estimand (International Council for Harmonisation, 2019). Everything else in the decomposition is scaffolding: the main effects describe the overall differences between protocols and the average time course, and the subject term accounts for between-person variation, useful for a faithful fit but not the quantity the study came to test.

A training study usually asks more than one question of this kind: whether HIIT beats control, whether MICT beats control, and which of the two protocols is better. Nothing changes. Each is one concrete comparison, read off the same fit and tested as though it stood alone (Schappacher-Tilp and Tilp, 2026). Whether the comparison appears in the output depends on how the model happens to be coded, and the overall tests the software prints, one for each term, do not directly report it. Standard packages will produce it on request, under headings such as contrasts or estimated marginal means (Lenth, 2016).

The same decomposition accommodates the variations a real study needs, one at a time. Adding continuous covariates such as age turns the model into an analysis of covariance, which adjusts the comparison without changing what is being compared. Fit it on a transformed scale, for a discrete outcome, and it becomes a generalised linear model. Give each participant their own random intercept, and often a random slope, and it becomes a mixed model. In each case, the model gains terms, but it is still only splitting the measurement into parts. None of the added terms becomes the question.

Naming the target comparison first, before any test is run, is the organising principle we are advocating. It fixes what “significant” is allowed to mean for this study, so that a result cannot be manufactured after the analysis by choosing which term to follow up, and it survives changes to the model: adding a covariate alters the machinery but not the question.

## What the old habit costs

A covariate-adjusted model makes this concrete with an example (**Figure 1**). A total of 60 participants are allocated to three arms, and each is measured every two weeks from baseline to week 12, with HIIT improving the most, MICT improving moderately, and Control staying flat. The comparison chosen in advance is how much faster HIIT climbs than Control, one of the questions set out above. We fit the same model twice, once when age, sex, and six further covariates carry no signal, and once when age predicts the outcome strongly. Because the data are simulated, we know the effect is real: we put it there, an advantage for HIIT of 3.0 mL kg^-1^ min^-1^ over the 12 weeks. In both fits, the overall test of whether the protocols follow different time courses misses it: in the fit with null covariates (*p* = 0*.*297) and in the fit with the age covariate (*p* = 0*.*444). The one comparison named in advance, read off each of those same two fits, finds it both times (*p* = 0*.*003 and *p* = 0*.*002).

**Figure 1:**
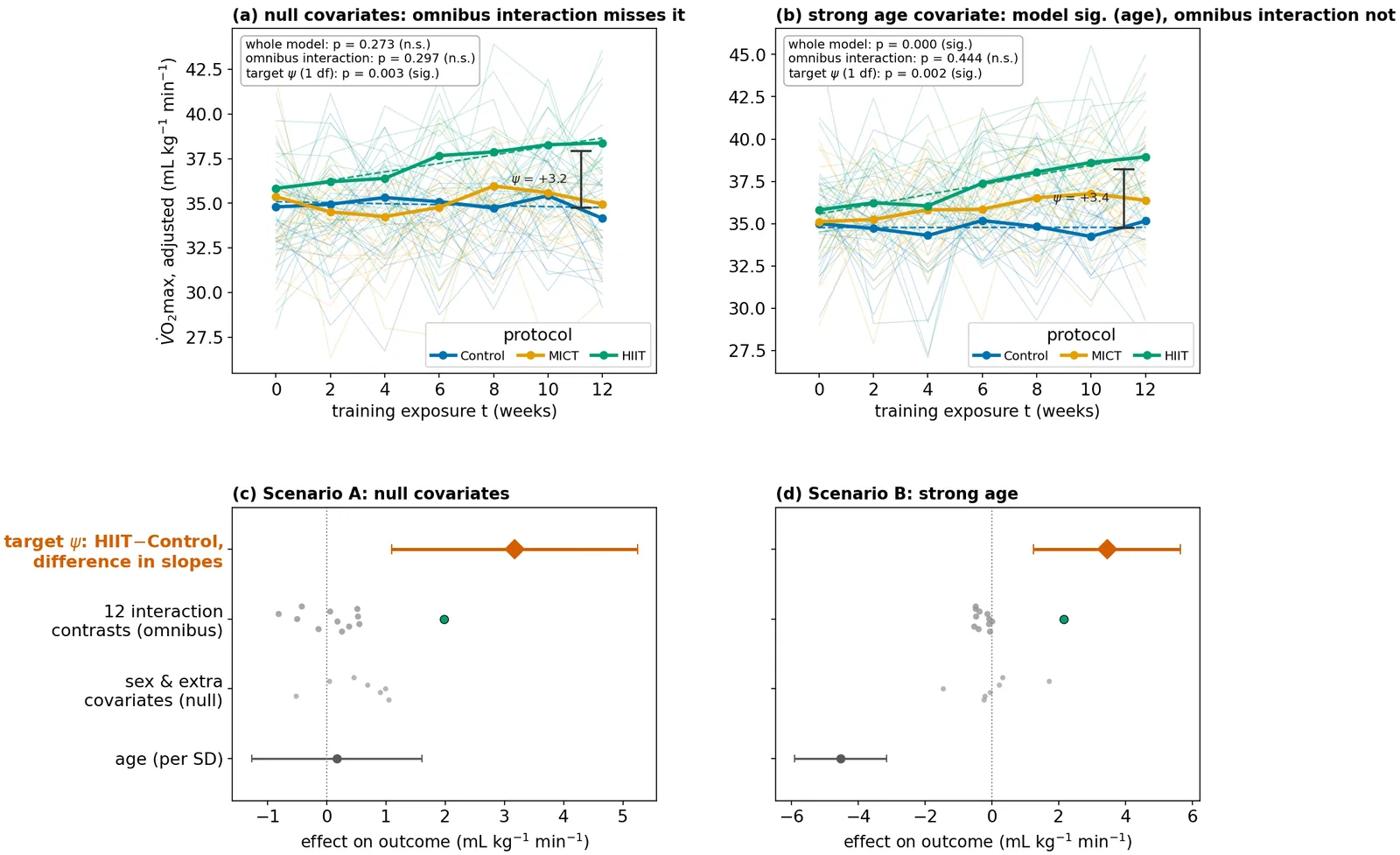
One covariate-adjusted fit, three questions. A total of 60 participants, 20 per arm, are assigned to Control, MICT, or HIIT and measured every two weeks from baseline to week 12, so each contributes seven measurements. Data were generated so that Control remains flat, MICT improves moderately, and HIIT improves the most, with each trajectory being approximately linearly (faint lines: individual participants, adjusted for the covariates and for their own level; dashed lines: fitted linear trends for Control and HIIT, whose slope difference is the target comparison *ψ*, generated at 3*.*0 mL kg^−1^ min^−1^). The model adjusts for age, sex, and six further covariates and gives each participant a random intercept. **(a)** With null covariates, the overall test of whether the protocols differ in their time courses is not significant (*p* = 0*.*297); however, the single planned comparison, the difference in slopes, is significant, consistent with the data-generating process (*p* = 0*.*003). The bracket marks *ψ*, the extra $\dot{V}O_{2}max$ that HIIT gains over Control across the 12 weeks. **(b)** With a strong age covariate, the test of the whole model, every term at once, is highly significant (*p<* 0*.*001), but that is driven by age, not training: the overall interaction is still not significant (*p* = 0*.*444), and only the planned comparison recovers the generated effect (*p* = 0*.*002). **(c, d)** The fitted comparisons from each fit: the target *ψ* stands clear of zero, whilst the 12 separate comparisons the overall test combines mostly hug zero, as do the null covariates. In panel **(d)**, age alone carries the whole model. Across 3,000 simulated studies, the planned comparison reaches significance in 79% of them, against 40% for the overall test (**Supplementary Section S4**).

Why does the overall test miss an effect that is plainly there? It has to cover every way three protocols could have differed across seven timepoints. Three protocols allow two independent comparisons, and seven timepoints allow six, so there are 12 separate ways in all. The study asked about one of them. Spreading the same evidence over 12 questions instead of one is what costs the power. In our simulations, testing the target comparison directly roughly doubles its power (it reaches significance in 79% of simulated studies here, against 40% for the overall test; **Supplementary Section S4**). The cost does not diminish as the outcome leaves the continuous world: for a discrete responder outcome measured repeatedly and fitted with a logistic model that allows the measurements from each participant to correlate (Liang and Zeger, 1986), the focused test again clearly outperforms the overall test (power of 72% against 41%, and 67% against 36% when the observations are independent). How big the gap is depends on the design: the fewer ways the overall test has to cover, the smaller the gap gets. However, its size is not the point. The gap is there only because the overall test is answering a question the study never asked, so it is not a cost to be kept small. It is one to avoid altogether.

## The recipe

Before collecting data, write down the one comparison the study is about, the scale on which it is claimed, and the form it takes. The comparison comes first. The scale and the form each require separate consideration; we come to them at the end of this section. Suppose we ask whether maximal oxygen uptake increases more under HIIT than under control over 12 weeks. The effect of interest is a single difference: the change under HIIT minus the change under Control, say a gain of 5*.*0 against 1*.*0 mL kg^−1^ min^−1^, a difference of 4*.*0. If there is nothing to adjust for, no model is needed at all: the difference is read straight from the group means and tested on its own. Bring in age and sex and the raw means are confounded, so you fit an analysis of covariance and read the same difference, now adjusted, off the fit. Either way, it is one quantity and one test, reported with its confidence interval, whatever the overall test says. If the question is instead at which weeks a protocol has pulled ahead, that is a search rather than a single question, what we called localisation above: test each week and control the false discovery rate (Benjamini and Hochberg, 1995; Schappacher-Tilp and Tilp, 2026).

### Mixed models

Participants frequently differ not only in where they start but also in how strongly they respond, so the model should give each of them their own slope and their own intercept. These random terms are bookkeeping for the design, not effects to test. **Figure 2** also shows the target comparison in a different form: not a difference in slopes but the HIIT-minus-Control gap at week 12 against baseline, read off the fixed effects, alongside the same gap at each earlier week. Again, the overall test misses what the planned comparison finds. Across 3,000 simulated studies, the planned comparison reaches significance in 68% of them, against 42% for the overall test (**Supplementary Section S4**).

**Figure 2:**
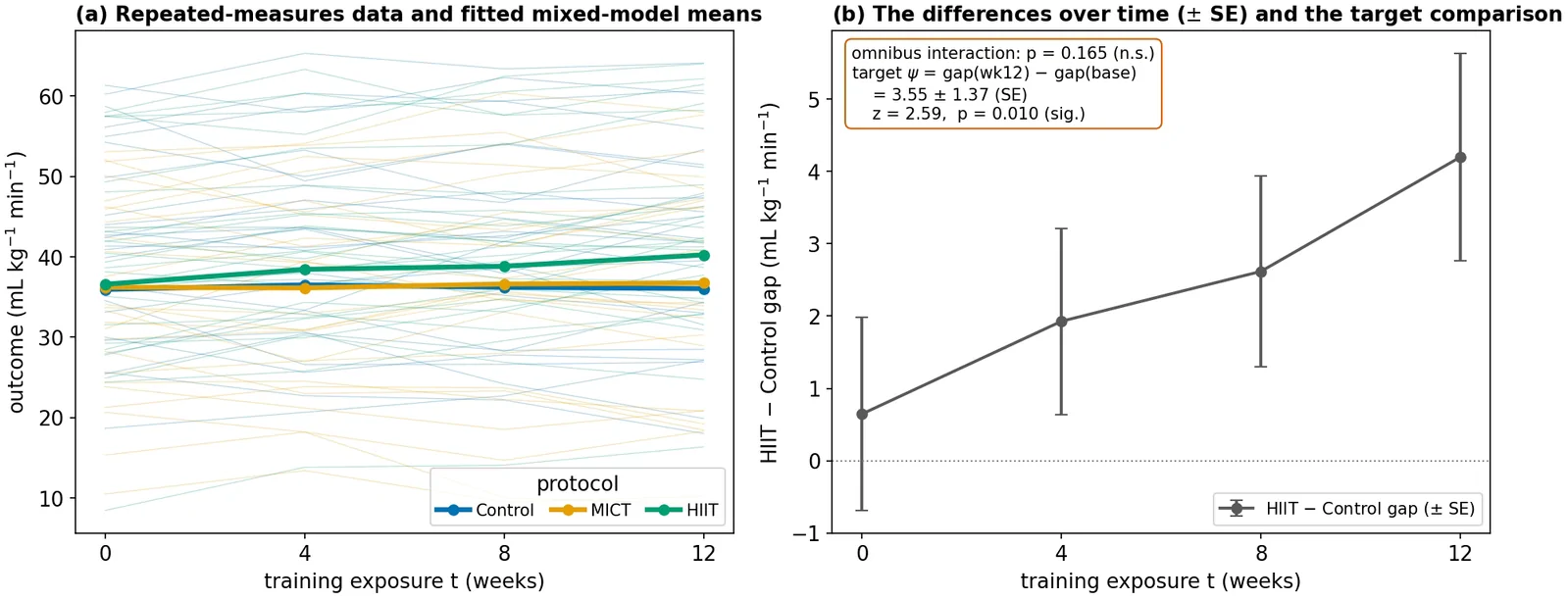
Letting participants respond differently changes the model, not the report. Participants in the Control, MICT, and HIIT arms are measured at 0, 4, 8, and 12 weeks, and the analysis adjusts for age and sex. Repeated measurements on the same person are related to one another, so each participant is given their own starting point and their own rate of change. What is reported is still one number. **(a)** Within-participant trajectories (faint) and the fitted mean profile for each arm (bold). **(b)** The target comparison, read from the same fit: the HIIT-minus-Control gap at each week (points ± SE), and its change from baseline to week 12, *ψ* = 3*.*55 ± 1*.*37 (SE), *p* = 0*.*010. The overall test from the same fit is not significant (*p* = 0*.*165); the planned comparison is.

### One step further for a discrete outcome

If the question is whether participants respond to training at all, rather than by how much, the effect can be stated as a difference between the two responder rates or as a ratio of them. Say MICT and HIIT both start with 10% of participants responding, and by week 12 MICT reaches 40% and HIIT reaches 70%: a difference of 30 percentage points, with an odds ratio of 3*.*5. However, the two do not vary together. In a squad where MICT brings 80% of players to responder status by week 12, rather than the 40% above, that same odds ratio puts HIIT at 93%, a lead of 13 points rather than 30 (Loftus, 1978; Ai and Norton, 2003; Norton et al., 2004; VanderWeele, 2015). Neither the difference nor the ratio is wrong. So decide in advance which of the two, the difference or the ratio, the study claims.

### Choosing the form before the data exist

Fixing the comparison in advance also means fixing its form. Range of motion after a stretching programme and strength in previously untrained participants may rise steeply for four to six weeks and then flatten. A difference in linear slopes, prespecified in good faith, may then measure something other than what the study intended: in our simulation of two plateauing arms, it is comfortably significant, yet indicates that the are still diverging at week 12 at approximately 12 times their actual rate (**Supplementary Figure S1**). So prefer the form that assumes the least. A difference between arms at a fixed time, with time entered as a factor, is valid whatever happens in between. Claim a difference in slopes only if linearity can be defended in advance or if the rate itself is the question.

## The number is only as good as the model

Everything above concerns which quantity to test. However, a number is only as reliable as the model it was read from, and a model that misdescribes the study will still print a confident answer to a question it was not built for. Five things are worth getting right.

- Measure covariates before randomisation. Adjusting for something recorded after the intervention began can bias the comparison itself (VanderWeele, 2015).
- Check that a covariate has the same influence on the outcome in every arm. If age predicts the gain under HIIT but not under Control, there is no single adjusted difference between the arms to report, because the difference now depends on the participant’s age. Say so and report the comparison at stated covariate values (Searle et al., 1980; Lenth, 2016).
- Let participants respond at different rates unless there is a reason not to. In training studies, they nearly always do, and a model that forces one common rate on everyone will too often declare an effect that is not there (Kenward and Roger, 1997).
- Plan for dropout. The participants who stop are usually the ones not responding, so say how you handle them and show that the conclusion survives a different assumption (International Council for Harmonisation, 2019).
- For discrete outcomes, check whether the data vary more than the model expects. A logistic or count model fixes in advance how much variation there should be around each predicted value; if the real data vary more than that, the standard errors are too small, and the comparison looks more certain than it is. Repeated measurements on the same participant have the same effect unless the model is told about them (Liang and Zeger, 1986).

## When an overall test is the right test

An overall test is the right test when the overall question is the one the study prespecified. We only object when an overall test is used in place of a specific question the study already had. We do not object when the overall question is itself the study’s question. The clearest case is screening. Six recovery modalities are compared with no prior favourite, and the study asks whether they differ at all before anyone commits to a particular comparison. The overall test is then the honest main result, and whatever follows it is exploratory. To decide whether the overall test is the right one, ask what the study was built to estimate. If it is a single comparison you could write down before seeing the data, test that comparison. If the target is a whole term, for example, whether several options differ at all or whether the protocols share a common time course, then an overall test is appropriate. If it is neither, the question is not yet precise, and sharpening it at the design stage will do more for the study than any choice of test.

## Does dropping the gate cost error control?

A natural objection is that dropping the overall gate gives up the error control it provided. In the designs this article is about, there is not much to give up. The familiar “protected” sequence, a significant overall *F* before unadjusted pairwise follow-ups (Fisher’s protected least significant difference), has a familywise guarantee only in simple cases and generally does not extend to more complex designs (Maxwell et al., 2018). Error control comes from declaring the family, and what the family is depends on what the study claims rather than on how many tests the software ran (Schappacher-Tilp and Tilp, 2026).

## Discussion

Our recommendation is easily summarised: fit the model the design demands, then base inference on the comparison the study was designed to make rather than on the table the software prints. This asks for a larger model, not a smaller one. Let it include every covariate, timepoint, and random effect the design needs, then read the one relevant number off the fit. Treating the overall test as a gate inverts the priorities: in our simulations, a real, prespecified effect is set aside because the overall test fails a test it was never meant to pass.

This is not a case against overall tests: they are the right tool for a genuinely global or exploratory question, and we have set out above when that is the case. We caution only against letting an overall test act as a gatekeeper for a prespecified question it was never asked to answer, or against splitting the data by group or timepoint when a single model and a single contrast answer it more powerfully (Searle et al., 1980; Lenth, 2016). Naming the comparison is not on its own enough, as the preceding section sets out, and none of this competes with design, as we said at the outset.

A statistical model is a tool for estimating meaningful quantities, not a menu of default tests. Taken seriously, that reshapes practice upstream: studies are planned around the target comparison, preregistrations name the contrast and its scale, and reviews ask whether that quantity was well estimated rather than whether an overall test reached significance. It also gives the replication literature something to work with, because a study that names one number has named the thing a replication would try to recover (Murphy et al., 2025; Kowalski, 2026). The overall test keeps its place for the global question it answers; it should not stand in for the one the investigator came to ask. Decompose freely, test tightly.

## Data Availability

The original contributions presented in the study are included in the article/**Supplementary Material**. Further inquiries can be directed to the corresponding author.
