# Supplementary material for: Fit one model, report one number: how to analyse a training or intervention study
Source: Front Physiol. 2026 Sep 18;17:1949947. doi: 10.3389/fphys.2026.1949947 (PMC13630658; doi:10.3389/fphys.2026.1949947)
Supplement: Supplementary file 1 [file DataSheet1.pdf]

Supplementary Material

Fit one model, report one number: how to analyse a training or intervention study

Gudrun Schappacher-Tilp [1] **and Markus Tilp** [2,*]

[1] *Department of Engineering, FH JOANNEUM - University of Applied Sciences, Graz, Austria*
[2] *Department of Human Movement Science, Sport and Health, University of Graz, Graz, Austria*

Correspondence*:
Markus Tilp
markus.tilp@uni-graz.at

S1 GLOSSARY AND NOTATION

The *estimand*, or target comparison, is the single number the study is designed to estimate, fixed by the scientific question and not by the model. A *contrast* is a weighted difference between group means expressing one specific comparison, for instance HIIT minus control. An *interaction* is a difference of differences: whether the change over time under one protocol exceeds the change under another. An *omnibus test* is a single test asking whether any part of a model term departs from zero, without saying which part. A *main effect* is the average difference between protocols, or the average time course, ignoring the other factor. A *family* is a set of comparisons the study's claim rests on jointly, so that a false claim on any one of them would compromise the conclusion; error control then applies across the set as a whole. Several pre-specified comparisons do not make a family merely by being several: three pairwise comparisons of protocols, each with its own conclusion, are three separate questions, and whether one holds does not bear on whether another does. The *familywise error rate* is the chance of at least one false positive across a family. The *false discovery rate* is the expected proportion of false positives among the comparisons declared significant. A *random effect* is a per-participant adjustment that absorbs the correlation between one person's repeated measurements.

Two-factor repeated-measures ANOVA.

Each observation y_{ijk}, for protocol i, timepoint j and participant k, is split into a benchmark and a sum of interpretable deviations,

$$y_{ijk} = \mu + \alpha_i + \tau_j + (\alpha\tau)_{ij} + s_k + \varepsilon_{ijk}, \tag{S1}$$

with grand mean μ, protocol deviation α_i, time deviation τ_j, interaction $(\alpha\tau)_{ij}$, participant deviation s_k, and residual ε_{ijk}.

The target comparison with time as separate timepoints.

If the question is whether HIIT raises $\dot{V}\mathrm{O}_2\mathrm{max}$ more than control from baseline (time 0) to week 12, the target comparison is the single interaction contrast

$$\psi = \big[(\alpha\tau)_{\mathrm{HIIT},12} - (\alpha\tau)_{\mathrm{HIIT},0}\big] - \big[(\alpha\tau)_{\mathrm{Ctrl},12} - (\alpha\tau)_{\mathrm{Ctrl},0}\big], \tag{S2}$$

the amount by which the gain under HIIT exceeds the gain under control. It is built entirely from the interaction terms: the grand mean and both main effects cancel in the difference of differences. With the numbers used in the main text, a gain of 5.0 under HIIT against 1.0 under control, $\psi = 4.0\,\mathrm{mL\,kg^{-1}\,min^{-1}}$.

Covariate adjustment and generalized linear models.

With covariates and time as a continuous trend,

$$g\big(\mathbb{E}[y]\big) = \beta_0 + \beta_{\text{age}}\,\text{age} + \beta_{\text{sex}}\,\text{sex} + (\beta + \gamma_p)\,t, \tag{S3}$$

where $\mathbb{E}[y]$ is the average outcome the model predicts for a given protocol, time, age and sex, and the link function g sets the scale on which these pieces add up. For an ordinary measurement such as $\dot{V}\mathrm{O_2max}$, g is the identity and the equation is read in the outcome's own units. For a yes/no outcome, g is the logit and the model works on a rescaled version of the responder percentage rather than on the percentage itself, which is the origin of the scale-dependence problem discussed in the main text. Each protocol p changes over time t at its own rate $\beta + \gamma_p$, and the target comparison is the difference between two of those rates,

$$\psi = \gamma_{p_1} - \gamma_{p_2}. \tag{S4}$$

A mixed model adds participant-specific random intercepts, and often random slopes, to absorb the correlation between one participant's repeated measurements. These random terms change the standard error of ψ but not its definition.

S2 INFERENTIAL SITUATIONS AND THE ERROR CONTROL EACH NEEDS

Table S1 sets out the three situations distinguished in the main text, together with the error rate each requires and the procedure that controls it. The argument for and against adjustment in each case is developed at length in our companion paper (Schappacher-Tilp and Tilp, 2026). Two points are worth drawing out. The first is that no row uses an omnibus test as a gate. Where the omnibus appears at all it is the analysis itself, in the row where the global question is the one the study asked. Error control comes from declaring the family and choosing the error rate, not from a preliminary global test. The second is that the number of comparisons is not what decides the matter. Several comparisons, each of which carries its own conclusion, are not a family and need no adjustment, however many of them there are, because no single claim rests on more than one of them and no error rate attached to any one of those claims is inflated. Three pairwise comparisons of training protocols are three separate questions. What creates a family is a claim that rests on the set jointly, rather than on a member the study named in advance. Localization, asking which of several timepoints or outcomes show an effect, is that same situation: the set is declared, the member to be claimed is not. Where control is called for, we take the false discovery rate as the default. It answers the question a declared set actually poses, what proportion of what I report is likely to be wrong, and it does not pay for that answer with the power that familywise procedures cost (Benjamini and Hochberg, 1995; Schappacher-Tilp and Tilp, 2026). Familywise control is reserved for the one case that needs it, where a single false claim anywhere in the set would compromise the conclusion.

S3 WHEN THE RESPONSE PLATEAUS: A NONLINEAR SCENARIO

The scenarios in the main text have approximately linear trajectories, so the difference in slopes is both a natural target comparison and the right one. This section shows what happens when it is not.

Table S1. What is fixed in advance determines what has to be controlled. The family of comparisons is fixed by the study's claim structure, not by the number of tests the software happened to run.

Situation	Fixed in advance	Error rate to control	Procedure	Omnibus gate?
Pre-specified target comparisons, each carrying its own conclusion, e.g. HIIT against Control, and separately MICT against Control	Each contrast, its scale and its direction	Type I error for each confirmatory claim separately	Test each at the nominal level. No adjustment, however many there are: no claim rests on more than one test, so no error rate is inflated	No
A declared set, where the claim is about the set rather than about a member named in advance, e.g. which of several timepoints show an effect, or which of several outcomes support the programme	The set, but not which member will be claimed	False discovery rate by default; familywise if one false claim would sink the conclusion	Benjamini-Hochberg over the declared set (Benjamini and Hochberg, 1995). Where a single false claim would compromise the conclusion, control the familywise error rate instead: Holm (Holm, 1979), or Dunnett for several arms against a common control (Dunnett, 1955).	No
A genuinely global question, e.g. do six recovery modalities differ at all	That the question is the whole term	Type I error for that one global claim	The omnibus test is the analysis, not a gate. Anything that follows it is exploratory	It is the test
Exploratory comparisons chosen after inspecting the data	Nothing	Not honestly controllable once the data have chosen the hypothesis	Report as exploratory, with effect sizes and intervals, and do not present as confirmatory. Adjustment does not repair selection	Irrelevant

Ankle dorsiflexion range of motion after a stretching programme, like strength in previously untrained participants, rises steeply for the first four to six weeks and then flattens. We simulate two arms, control and stretching, measured every two weeks for twelve weeks with 24 participants per arm. Both arms approach a plateau exponentially, with the same time constant but different asymptotes, so the arms differ in where they end up and not in an average rate of change. Parameters are in Table S2. The asymptotic difference between the arms is $4.0°$, of which 98% has arrived by week 12, so the true difference between arms at week 12 is $3.93°$.

We analyse the same data three ways: the omnibus arm-by-time interaction on six degrees of freedom, the difference in linear slopes, and the difference between arms at week 12. Both single-degree-of-freedom contrasts come from the same kind of fit as in the main text, the first from a linear-in-time model and the second from a model saturated in time; participants enter as fixed intercepts, which for within-participant time contrasts in a balanced complete design is equivalent to a random-intercept mixed model.

Statistical power.

Over 4000 replicates, power at $\alpha = 0.05$ was 0.88 for the omnibus interaction, 0.94 for the difference in linear slopes, and 0.89 for the difference at week 12.

The failure is descriptive.

A difference in linear slopes forces the between-arm gap to be a straight line through the origin, and on these data that misdescribes the study twice over (Figure S1). It understates the difference achieved by week 12, implying $3.51°$ against a true $3.93°$. More seriously, it reports the two arms as still diverging at week 12 at $0.29°$ per week, when the true rate at that point is $0.024°$ per week, an overstatement by a factor of twelve, because a straight line cannot flatten.

The remedy is to choose the target comparison from the expected shape of the response rather than from a template: here the difference between arms at week 12, or equivalently the difference in plateau, and in other settings the area under the curve or the time to reach a nominated threshold. This is the same requirement as the model-specification checks in the main text, applied to the mean structure.

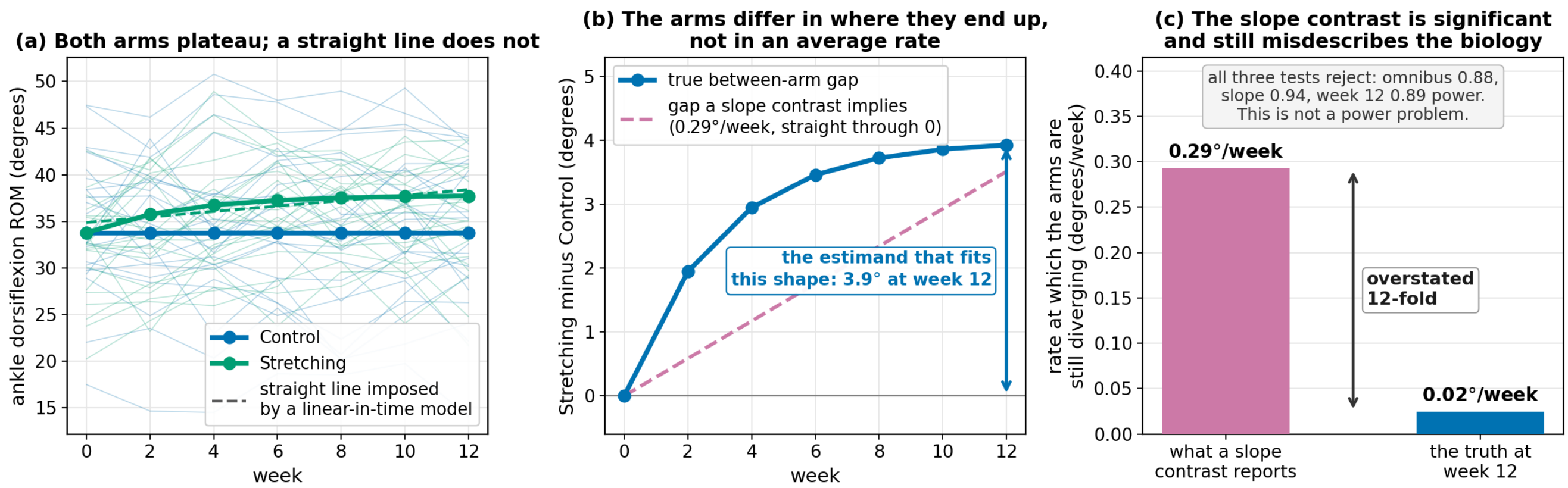

Figure S1. A pre-specified contrast can be well powered and still describe the study wrongly. (a) Simulated ankle dorsiflexion range of motion for control and stretching arms (faint lines: individual participants; bold: true mean trajectories). Both arms plateau; the dashed lines are the straight lines a linear-in-time model imposes on them. **(b)** The true between-arm gap week by week (solid) against the gap a slope contrast implies, a straight line through the origin (dashed). The arms differ in where they end up, $3.9°$ at week 12, not in an average rate. **(c)** All three analyses reject comfortably, so this is not a power problem. The failure is that the slope contrast reports the arms as still diverging twelve times faster than they are.

Simulation parameters

Table S2 lists the parameters of the nonlinear scenario. The outcome, ankle dorsiflexion range of motion after a plantar-flexor stretching programme, was chosen because its magnitudes and its variability are well documented in our own laboratory. Values are taken from Konrad and Tilp (2014), a six-week randomised stretching intervention in which dorsiflexion range of motion rose from $33.8 \pm 6.3°$ to $37.8 \pm 7.2°$ in the stretching arm and did not change in the control arm, and from Konrad et al. (2024), whose arm sizes are close to those simulated here. The two remaining quantities, the residual standard deviation and the time constant, are modelling choices rather than measured values, for the reason given below the table.

Table S2. Parameters of the nonlinear plateau scenario (Figure S1).

Quantity	Value	Basis
Design	2 arms × 7 timepoints	measured every 2 weeks to week 12
Participants per arm	24	48 randomised to two arms in Konrad and Tilp (2014); 18 and 15 per arm in Konrad et al. (2024)
Baseline range of motion	33.8°	as reported by Konrad and Tilp (2014)
Eventual gain, control	0.0°	no significant change in the control arms of Konrad and Tilp (2014) or Konrad et al. (2024)
Eventual gain, stretching	4.0°	4.0° over six weeks in Konrad and Tilp (2014); a plateau, so a twelve-week programme reaches the same level
Between-participant SD	6.3°	as reported at baseline by Konrad and Tilp (2014); 6.7° and 8.0° in Konrad et al. (2024)
Time constant of the approach to plateau	3 weeks	modelling choice: about 95% of the gain is reached by week 9
Within-participant residual SD	3.0°	modelling choice
Replicates	4000	
Nominal level	$\alpha = 0.05$	

We are explicit that the last two rows are not measured quantities. Stretching trials almost always report only a pre-test and a post-test, so the shape of the approach to the plateau is rarely observed, and the within-participant residual standard deviation is not separable from the between-participant standard deviation in a two-timepoint design.

S4 PARAMETERS AND POWER OF THE SIMULATIONS IN THE MAIN TEXT

The main text quotes power figures for two simulated settings, a continuous outcome and a yes/no outcome. This section gives the parameters of each, the power each analysis achieved, and the calculation behind the statement that the same data can show an interaction on one scale and none on another. Every scenario is a Monte Carlo experiment with a fixed seed. Sample sizes and effect sizes were chosen to sit inside the ranges reported for the training literature, as noted in the tables.

Continuous outcome: the covariate-adjusted repeated-measures model of Figure 1.

Three arms, Control, MICT and HIIT, with 20 participants each, are assessed every two weeks from baseline to week 12, so every participant contributes seven measurements and the study has 60 participants and 420 observations. Time enters as a factor, which is what makes the protocol-by-time interaction carry twelve degrees of freedom. The model adjusts for age, sex and six further covariates and gives each participant a random intercept. Parameters are in Table S4.

Three verdicts are read from the same fit: the whole-model test on all fixed effects, the omnibus protocol-by-time interaction on twelve degrees of freedom, and the pre-specified target comparison, the difference in linear trends between HIIT and Control expressed over the twelve weeks. Over 3000 replicates at $\alpha = 0.05$:

Table S3. Power of the three tests read from one covariate-adjusted repeated-measures fit (Figure 1). The target comparison is the pre-specified one; the other two are what the software prints by default. The final column is the proportion of replicates in which a study that had gated on the omnibus interaction would have discarded a target comparison that was in fact significant.

Scenario	Whole model	Omnibus interaction	Target comparison	Target significant, omnibus not
A: null covariates	0.719	0.397	**0.789**	0.407
B: strong age covariate	0.999	0.394	**0.790**	0.412

The target comparison is recovered about twice as often as the omnibus interaction, and in scenario B the whole-model test is significant in essentially every replicate although no covariate carries any training information: that significance is age. This is the dissociation the main text describes.

Table S4. Parameters of the continuous-outcome scenario (Figure 1).

Quantity	Value	Basis
Design	3 arms $\times$ 7 timepoints, repeated measures	assessed every 2 weeks to week 12; every participant measured at every timepoint
Participants per arm	20 (60 in total)	within the usual range for a three-arm training trial
Baseline $\dot{V}\mathrm{O_2max}$	$35\,\mathrm{mL\,kg^{-1}\,min^{-1}}$	typical for previously untrained adults
True target comparison ψ	$3.0\,\mathrm{mL\,kg^{-1}\,min^{-1}}$	the HIIT minus Control gain over 12 weeks; MICT is set to half of it, Control flat
Between-participant SD	$5.0\,\mathrm{mL\,kg^{-1}\,min^{-1}}$	typical between-person spread at baseline
Within-participant SD	$3.0\,\mathrm{mL\,kg^{-1}\,min^{-1}}$	repeated assessment over weeks, so biological variation as well as measurement error
Covariates	age, sex and 6 further	all null in scenario A; in scenario B age acts at $-0.45\,\mathrm{mL\,kg^{-1}\,min^{-1}}$ per year, with age SD 10 years
Replicates	3000	
Nominal level	$\alpha = 0.05$	

Continuous outcome: the mixed model of Figure 2.

The second continuous scenario uses four timepoints rather than seven, 25 participants per arm, and a per-participant random slope as well as a random intercept, so the protocol-by-time interaction carries six degrees of freedom instead of twelve. The target comparison is the change in the HIIT minus Control gap from baseline to week 12. Over 800 fits at $\alpha = 0.05$ the target comparison was recovered in 68.2% of replicates against 41.7% for the omnibus interaction, and in 28.6% of replicates the target was significant while the omnibus was not. The gap is smaller than in Figure 1, as the smaller number of interaction degrees of freedom would lead one to expect.

Yes/no outcome.

A responder indicator is analysed in a 3×4 protocol-by-time layout, with the target comparison the change in the HIIT minus MICT gap from baseline to week 12 and the omnibus interaction carrying six

degrees of freedom. We ran it twice. In the repeated-measures version, which is the one the main text quotes, 30 participants per protocol (90 in total) are each measured at all four timepoints, their responses are correlated through a participant random intercept, and the marginal model is fitted by generalized estimating equations with an exchangeable working correlation and a robust sandwich covariance (Liang and Zeger, 1986). In the independent-observations version each of the twelve cells contains a separate group of 40 participants and the saturated logistic model has a closed-form fit. Parameters and power are in Table S5. In both versions the row at zero interaction reports the achieved Type I error, which sits near the nominal level, so the comparison between the two tests is a fair one.

Table S5. Parameters and power of the yes/no scenarios. Power is the rejection rate at $\alpha = 0.05$. The interaction is placed entirely in the single pre-specified direction on the logit scale, so the omnibus test is spending five of its six degrees of freedom on directions in which nothing is happening.

	**Repeated measures (GEE)**	**Independent observations**
Design	3 protocols $\times$ 4 timepoints	3 protocols $\times$ 4 timepoints
Participants	30 per protocol (90 total), each measured 4 times	40 per cell (480 total), each measured once
Baseline responder rate	0.40	0.40
Responder rate range across cells	0.40 to 0.73	0.40 to 0.73
Within-participant correlation	random intercept SD 0.80 on the logit scale	none
Interaction on the logit scale	2.0	1.6
Estimation	GEE, exchangeable, robust SE	saturated logistic, Wald
Replicates	1000	3000
Type I error, target test	0.052	0.048
Type I error, omnibus test	0.066	0.042
**Power, target comparison**	**0.715**	**0.669**
**Power, omnibus interaction**	**0.407**	**0.358**

The same effect, two scales, two magnitudes.

The main text states that a discrete effect has to be reported with the scale it is claimed on. The demonstration is arithmetic rather than statistical, and needs no sampling.

In a randomised design both arms start from the same responder rate, $p_{\mathrm{HIIT},0} = p_{\mathrm{MICT},0}$. The baseline terms then cancel on *every* scale, so

$$\psi_{\mathrm{logit}} = 0 \iff p_{\mathrm{HIIT},12} = p_{\mathrm{MICT},12} \iff \psi_{\mathrm{risk}} = 0. \tag{S5}$$

With equal baselines the two scales therefore always agree on whether there is an interaction. A construction in which one scale shows no interaction and the other does requires the arms to differ at baseline, which randomisation is there to prevent.

What the scales do not agree on is the magnitude of the effect, and where that magnitude transfers. Let both arms start at 10% responders and reach 40% under MICT and 70% under HIIT at week 12. Then

$$\psi_{\mathrm{risk}} = 0.300, \qquad \mathrm{OR} = \frac{0.70/0.30}{0.40/0.60} = 3.50. \tag{S6}$$

Applying that same odds ratio to populations with other MICT rates gives the differences in Table S6. The ratio is the quantity that carries over; the difference in percentage points does not, and has to be quoted with the baseline rate it was measured from (Ai and Norton, 2003; Norton et al., 2004; VanderWeele, 2015).

Table S6. One odds ratio, many differences. The odds ratio is held at 3.50, the value implied by the trial above. The HIIT rate and the difference follow arithmetically from the MICT rate.

MICT responder rate	Implied HIIT rate	Difference (percentage points)
0.20	0.467	26.7
0.40	0.700	30.0
0.60	0.840	24.0
0.80	0.933	13.3
0.90	0.969	6.9

The size of the gap is a property of the design.

The advantage of the target comparison over the omnibus test grows with the number of degrees of freedom the omnibus has to spread itself over. It is largest in the continuous-outcome scenario above, where seven timepoints entered as a factor give the interaction twelve degrees of freedom, smaller in the yes/no scenarios with six, and almost absent in the plateau scenario of Section S3, where the omnibus reaches 0.88 against 0.94 for the difference in linear slopes. The case for testing the target comparison does not rest on the size of that gap. It rests on the gap being avoidable, and on the omnibus answering a question the study did not ask.

S5 GENERATED DATA

The simulated datasets are provided as comma-separated value files, one row per participant per visit:
Data Sheet 2.csv (Figure 1: ANCOVA scenario A, null covariates),
Data Sheet 3.csv (Figure 1: ANCOVA scenario B, strong age covariate),
Data Sheet 4.csv (Figure 2: mixed repeated measures), and
Data Sheet 5.csv (Figure S1: nonlinear plateau).

REFERENCES

Ai, C. and Norton, E. C. (2003). Interaction terms in logit and probit models. *Economics Letters* 80, 123–129

Benjamini, Y. and Hochberg, Y. (1995). Controlling the false discovery rate: a practical and powerful approach to multiple testing. *Journal of the Royal Statistical Society, Series B* 57, 289–300

Dunnett, C. W. (1955). A multiple comparison procedure for comparing several treatments with a control. *Journal of the American Statistical Association* 50, 1096–1121. doi:10.1080/01621459.1955.10501294

Holm, S. (1979). A simple sequentially rejective multiple test procedure. *Scandinavian Journal of Statistics* 6, 65–70

Konrad, A., Reiner, M. M., Manieu, J., Fischer, J., Schöpflin, A., Tilp, M., et al. (2024). Seven weeks of pectoralis muscle stretching does not induce non-local effects in dorsiflexion ankle range of motion. *European Journal of Sport Science* 24, 259–265. doi:10.1002/ejsc.12061

Konrad, A. and Tilp, M. (2014). Effects of ballistic stretching training on the properties of human muscle and tendon structures. *Journal of Applied Physiology* 117, 29–35. doi:10.1152/japplphysiol.00195.2014

Liang, K.-Y. and Zeger, S. L. (1986). Longitudinal data analysis using generalized linear models. *Biometrika* 73, 13–22

Norton, E. C., Wang, H., and Ai, C. (2004). Computing interaction effects and standard errors in logit and probit models. *The Stata Journal* 4, 154–167

Schappacher-Tilp, G. and Tilp, M. (2026). To adjust or not to adjust? *Frontiers in Physiology*

VanderWeele, T. J. (2015). *Explanation in Causal Inference: Methods for Mediation and Interaction* (New York: Oxford University Press)
